# Factors Affecting Proximal Colon Cleansing Based on Bowel Movement Kinetics: A Prospective Observational Study

**DOI:** 10.1155/2019/7032971

**Published:** 2019-03-03

**Authors:** Dae Bum Kim, Kang-Moon Lee, Sung-Goo Kang, Sung Hoon Jung

**Affiliations:** ^1^Department of Internal Medicine, St. Vincent's Hospital, College of Medicine, The Catholic University of Korea, Suwon, Republic of Korea; ^2^Department of Family Medicine, St. Vincent's Hospital, College of Medicine, The Catholic University of Korea, Suwon, Republic of Korea; ^3^Department of Internal Medicine, St Paul's Hospital, College of Medicine, The Catholic University of Korea, Seoul, Republic of Korea

## Abstract

**Background:**

Previous studies have suggested that relatively poor bowel preparation in the proximal colon, compared to that in the distal colon, could decrease the usefulness of colonoscopy. The aim of this study was to determine whether the “first defecation time” after polyethylene glycol (PEG) administration affects the cleansing quality in the proximal colon.

**Methods:**

A total of 425 individuals who were scheduled to undergo a screening colonoscopy were enrolled prospectively at the healthcare center of St. Vincent's Hospital, Suwon, Korea, between April 2015 and March 2016. Bowel cleansing was performed using 4 L of PEG. Surveys were conducted to obtain information regarding the “first defecation time.” Endoscopists assessed the quality of bowel preparation in each bowel segment.

**Results:**

We investigated 425 consecutive eligible cases. The mean “first defecation time” after PEG administration was 54.35 min. The quality of bowel preparation was poorer in the proximal colon than that in the distal colon. The adequate (excellent, good) and inadequate (fair, poor) proximal colon preparation groups comprised 360 (84.7%) and 65 (15.3%) patients, respectively. A multivariate analysis revealed that female gender (*P* = 0.029), small waist circumference (*P* = 0.027), and the long “first defecation time” (*P* = 0.034) were independently associated with inadequate bowel preparation in the proximal colon.

**Conclusion:**

Our data document that the “first defecation time,” female gender, and a small waist circumference affect the quality of preparation in the proximal colon. Inadequate preparation in the proximal colon was more common in females. Patients with these factors undergoing colonoscopy should be monitored carefully.

## 1. Introduction

Colonoscopy is practiced worldwide for early detection of colon cancer and has lowered the death rate of colorectal cancer [1, 2]. The effectiveness of colonoscopy depends on the quality of bowel preparation. Colonoscopy is reportedly less effective in the proximal colon compared with the distal colon [3, 4], possibly due to relatively poor bowel preparation in the proximal colon [5, 6]. Adequate bowel preparation improves the detection rate of colonic lesions and renders colonoscopy technically straightforward [7, 8].

The factors associated with inadequate bowel preparation include old age, constipation, diabetes, dementia, stroke, and use of tricyclic antidepressants [9, 10]. However, the reported risk factors vary. Furthermore, few studies have evaluated proximal colon cleansing or the relationship between right colon cleansing and bowel movements [11, 12].

To our knowledge, the effect of bowel movement, particularly the first defecation time, on the degree of bowel preparation has not been investigated. Thus, we investigated the factors that affect bowel preparation in the proximal colon and determined whether the first defecation time after polyethylene glycol (PEG) administration affects cleansing quality in the proximal colon.

## 2. Methods

### 2.1. Patients and Study Design

This prospective observational study was conducted at the Comprehensive Medical Examination Center of St. Vincent's Hospital, Suwon, Korea. The study was approved by the Institutional Review Board at The Catholic University of Korea (VC150ISI0011). Written informed consent was obtained from all patients.

Consecutive patients who were scheduled for screening colonoscopy at the Comprehensive Medical Examination Center of St. Vincent's Hospital were enrolled prospectively. The exclusion criteria were advanced colon cancer, inflammatory bowel disease, previous surgical resection of the colon, and other comorbidities that can affect bowel movement.

Before the procedure, all patients were educated by nurses with verbal and printed information regarding the bowel preparation protocol. Patients were instructed to avoid eating a high-fiber diet for 3 days prior to the colonoscopy and to consume a clear liquid diet for lunch and dinner on the day before the examination, with no breakfast on the day of the exam. The preparation was ingested, beginning at 6:00 am. Participants were instructed to take 4 L of PEG (Colyte; Taejoon Pharma, Seoul, Korea) divided into 500 mL volumes, every 30 minutes until completion. All subjects ingested PEG at the Comprehensive Medical Examination Center. The coordinator recorded the times at which PEG ingestion started and finished, as well as the first defecation time, i.e., the interval between ingestion and the first excretion. After the patients confirmed that they had excreted cleanly, endoscopy procedures were carried out by expert endoscopists who had performed more than 1,000 colonoscopies.

The cleanliness of each bowel segment (proximal, cecum, and ascending colon; transverse, including the hepatic and splenic flexures; and left, from the descending colon to the rectum) was assessed by expert endoscopists. Each segment was assigned a score on a 4-point scale, defined as follows: 3: “Excellent,” entire mucosa of the colon segment seen well, with no residual staining, small fragments of stool, or opaque liquid; 2: “Good,” minor amount of residual staining, small fragments of stool, and/or opaque liquid, but mucosa of the colon segment is seen well; 1: “Fair,” portion of mucosa of the colon segment, but other areas of the colon segment are not well seen because of staining, residual stool, and/or opaque liquid; and 0: “Poor,” the unprepared colon segment with mucosa not seen because of solid stool that cannot be cleared [13, 14].

Demographic features and medical histories were reported by the patients. The characteristics of polyps and colonoscopic findings were obtained from pathology and colonoscopy reports. We defined the polyp and adenoma detection rates as the proportions of patients in whom more than one polyp and adenoma were detected.

### 2.2. Statistical Analyses

Continuous data are presented as mean ± SD. The *t*-test and the chi-square test were used to evaluate differences between variables, between the two groups in the univariate analysis. Variables that were significant or showed a tendency to be different in the univariate analysis were included in binary logistic regression for the multivariate analysis. SPSS software (SPSS Statistics 21 Standard for Medical Service; SPSS Inc., Chicago, IL) was used for all analyses. A *P* value of <0.05 was considered indicative of statistical significance.

## 3. Results

### 3.1. Patient Demographics and Bowel Movement Kinetics

**Figure 1:**
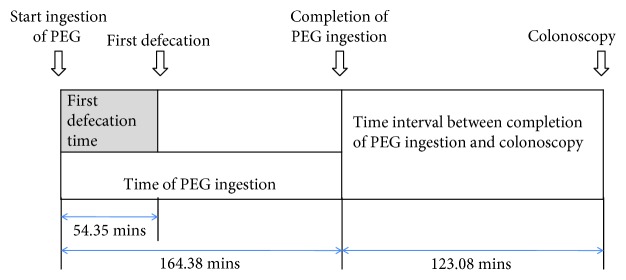
Summary of time intervals after PEG administration including the first defecation time.

**Figure 2:**
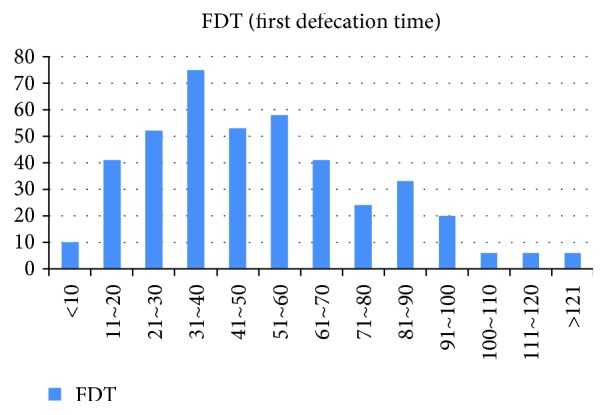
Minutes to the first defecation time.

A total of 425 consecutive eligible patients were enrolled in the study; their mean age was 49.1 ± 10.3 years, and 293 (68.9%) were males.  Figure 1 shows the average first defecation time, time to completion of PEG ingestion, and interval between PEG ingestion and colonoscopy. The first defecation time after ingestion of PEG ranged from less than 10 min to more than 120 min; the mean time was 54.35 min (Figure 2).

### 3.2. Bowel Preparation and Colonoscopy Results

**Figure 3:**
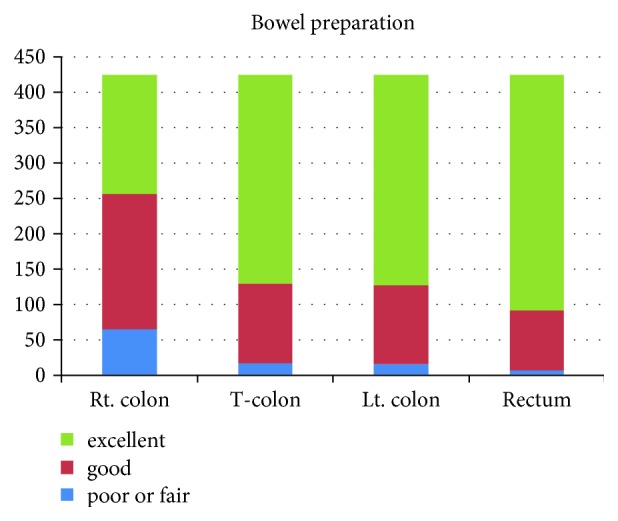
The degree of bowel preparation in each bowel segment.

Cecal intubation was performed for all procedures; the mean cecal intubation time was 4.1 ± 2.6 min. Polyps were endoscopically discovered in 194 patients, and adenomatous polyps were pathologically diagnosed in 31 patients. The quality of bowel preparation in the proximal colon was inferior to that in the distal colon (Figure 3).

### 3.3. Factors Associated with Inadequate Preparation in the Right Colon

The adequate (excellent, good) and inadequate (fair, poor) proximal colon preparation groups comprised 360 (84.7%) and 65 (15.3%) patients, respectively. The mean total PEG ingestion time was not different between the two groups (167.7 ± 30.7 vs. 163.8 ± 23.9 min, *P* = 0.337), but the mean first defecation time was longer in the inadequate group than in the adequate group (63.2 ± 31.8 vs. 52.8 ± 25.5, *P* = 0.015). Females were significantly more likely to have inadequate bowel preparation (*P* = 0.002). The mean body mass index (BMI) was not different between the two groups (24.0 ± 3.3 vs. 24.2 ± 3.2, *P* = 0.647); however, the mean waist circumference was significantly smaller in the inadequate group than in the adequate group (82.4 ± 7.6 vs. 85.6 ± 8.2, *P* = 0.004). The mean cecal intubation time was nonsignificantly longer in the inadequate group versus the adequate group (4.5 ± 2.9 vs. 4.1 ± 2.6, *P* = 0.187). The polyp and adenoma detection rates were not different between the two groups (Table 1).

In a multivariate regression analysis, female gender (OR, 1.892; 95% CI, 1.07–3.35; *P* = 0.029), small waist circumference (OR, 0.962; 95% CI, 0.93–0.99; *P* = 0.027), and the long first defecation time (OR, 1.011; 95% CI, 1.00-1.02; *P* = 0.034) were significantly associated with inadequate bowel preparation in the proximal colon (Table 2).

## 4. Discussion

The degree of bowel preparation is the most important factor impacting the quality of colonoscopy and could be affected by bowel movements and the interval between bowel preparation and colonoscopy. We aimed to identify factors that affect bowel preparation in the proximal colon and to assess the relationship between bowel movement kinetics and bowel preparation. To our knowledge, there are no data on the first defecation time after starting ingestion of PEG, and no study of bowel preparation has taken into consideration the first defecation time.

Colonoscopy is less effective in colorectal cancer in the proximal compared with the distal colon [4, 15, 16], possibly due to inadequate bowel preparation in the proximal colon. However, few studies have investigated the factors associated with poor preparation in the proximal colon.

In this study, the bowel preparation in the right colon was inferior to that in the distal colon, which was associated with female gender, small waist, and a longer first defecation time.

Half of the patients had a bowel movement within 1 h, and the mean first defecation time was 54.35 min. This finding is similar to a previous report of bowel preparation-induced bowel movement kinetics [8]. However, that study used PEG electrolyte lavage solution containing ascorbic acid (PEG-ELS+asc) (MoviPrep; Salix Pharmaceuticals, Raleigh, NC). In addition, we did not rely on patient reporting of the first defecation time. Inadequate preparation in the right colon was associated with a longer first defecation time, possibly due to slow bowel kinetics, e.g., constipation. Constipation is reportedly associated with poor bowel preparation [17, 18]. Although no study has evaluated the first defecation time during bowel preparation in constipated patients, the first defecation is delayed after laxative ingestion in patients with constipation [19]. Therefore, patients with slow bowel kinetics likely have a longer delay to the first defecation time and poor bowel preparation, especially in the right colon. We excluded patients with diseases (e.g., diabetes), as well as those taking medications that could affect bowel movements. We demonstrated that long fecal defecation time suggests inadequate bowel preparation in the proximal colon. Thus, in these patients with a long first defecation time, we recommend activities that can increase bowel movement or a long runway time from the last intake for the purge to the procedure.

Male gender is reportedly a predictor of inadequate bowel preparation [17, 20–22]. This is in contrast to our finding that females were significantly more likely to have inadequate bowel preparation. These gender differences are usually explained by different attitudes to healthcare and differences in the rate of adherence to bowel preparation between males and females [23]. However, all participants in this study were educated and performed bowel preparation in the hospital. In addition, previous studies evaluated the preparation status of the entire bowel, instead of each segment, and included patients with comorbidities, as well as those taking medications that could affect bowel kinetics. A recent large cohort study reported that females had a high rate of missing colorectal cancer after negative colonoscopy, and the missed colorectal cancer was most frequently in the proximal colon [24]. Our finding that inadequate preparation in the right colon is more common in females may explain this result.

Interestingly, inadequate bowel preparation in the right colon was related to a small waist, but not to BMI. A smaller waist circumference is associated with a longer cecal insertion time [25]. This may in turn be related to a smaller abdominal cavity, which leads to an acute bended colon, constipation, and delayed PEG excretion. Therefore, individuals with a small waist are more likely to exhibit inadequate bowel preparation in the right colon. This finding is in contrast to the results of Rotondano et al. [22] who found that male gender, higher BMI, chronic constipation, and runway time > 6 hours were associated with inadequate bowel cleansing of the right colon. However, the patients enrolled in that study were very heterogeneous, which means diverse indications, inpatients or outpatients, and variable comorbidities. These heterogeneities may have affected the outcome of the study, so further studies are needed.

Considering the results of this study, that women with a thin waist had an inadequate bowel preparation in the proximal colon, clinicians should recommend activities that can increase bowel movement or long runway times from the last intake for the purge to the procedure.

The strength of this study was that all subjects ingested PEG at the Comprehensive Medical Examination Center and all indicators, including the first defecation time, were determined by researchers. Furthermore, we excluded patients taking medications, as well as those with conditions that could affect bowel kinetics. This study also had several limitations. First, relatively few female subjects were enrolled. Second, the subjects were younger, and the ADR was lower, than in previous reports. Compared with a recent study [26], the subjects enrolled in our study were younger by 10 years and their indication for colonoscopy was screening. Therefore, the adenoma and polyp detection rates would be low and there would be no difference between the two groups. Third, we did not survey the bowel habits of the subjects, which could lead to selection bias.

In conclusion, female gender, small waist, and a long first defecation time were associated with inadequate bowel preparation in the right colon. Clinicians should be aware of this before colonoscopy and should check the clarity of the rectal effluent of females with a small waist and a longer first defecation time. However, further studies of the effect of controlling the first defecation time on bowel cleansing are needed. Moreover, methods of improving bowel clearance in patients with slow bowel movements should be developed.

## Figures and Tables

**Table 1:** Characteristics and univariate analysis of the adequate preparation group and inadequate preparation group in the proximal colon.

	Total	Adequate group	Inadequate group	*P* value
	(*n* = 425)	(*n* = 360)	(*n* = 65)	
Sex				0.002
Male (%)	293 (68.9)	259 (71.9)	34 (52.3)	
Female (%)	132 (31.1)	101 (28.1)	31 (47.7)	
Age (mean ± SD, years)	49.1 ± 10.34	49.2 ± 10.4	48.0 ± 10.3	0.392
BMI (kg/m^2^)	24.2 ± 3.2	24.2 ± 3.2	24.0 ± 3.3	0.647
Waist circumference (cm)	85.1 ± 8.2	85.6 ± 8.2	82.4 ± 7.6	0.004
First defecation time (min)	54.4 ± 26.7	52.8 ± 25.5	63.2 ± 31.8	0.015
Time of PEG ingestion (min)	164.4 ± 25.0	163.8 ± 23.9	167.7 ± 30.7	0.337
Time interval between completion of PEG ingestion and colonoscopy (min)	123.1 ± 30.8	123.3 ± 30.6	122.0 ± 31.7	0.765
Cecal intubation time (min)	4.1 ± 2.6	4.1 ± 2.6	4.5 ± 2.9	0.187
Adenoma detection rate (%)		7.5	6.1	0.701
Polyp detection rate (%)		46.4	41.5	0.470

**Table 2:** Independent factors associated with inadequate preparation of the proximal colon on multivariate analysis.

	Estimated value	Standard error	Odds ratio	95% CI^∗^	*P* value
Sex	0.638	0.291	1.892	1.07-3.35	0.029
Waist circumference	-0.039	0.018	0.962	0.93-0.99	0.027
First defecation time	0.011	0.005	1.011	1.00-1.02	0.034
Cecal intubation time	0.016	0.052	1.016	0.92-1.12	0.763

## Data Availability

The data used to support the findings of this study are available from the corresponding author upon request.
